# Impacts of Satellite Orbit and Clock on Real-Time GPS Point and Relative Positioning

**DOI:** 10.3390/s17061363

**Published:** 2017-06-12

**Authors:** Junbo Shi, Gaojing Wang, Xianquan Han, Jiming Guo

**Affiliations:** 1 School of Geodesy and Geomatics, Wuhan University, Wuhan 430079, China; wanggaojing@whu.edu.cn (G.W.); jmguo@sgg.whu.edu.cn (J.G.); 2 State Key Laboratory of Information Engineering in Surveying, Mapping and Remote Sensing, Wuhan University, Wuhan 430079, China; 3 Key Laboratory of Precise Engineering and Industry Surveying, National Administration of Surveying, Mapping and Geoinformation, Wuhan University, Wuhan 430079, China; 4 Engineering Safety and Disaster Prevention Department, Yangtze River Scientific Research Institute, Wuhan 430010, China; hanxq@mail.crsri.cn

**Keywords:** satellite orbit impact, satellite clock impact, real-time positioning, point positioning, relative positioning

## Abstract

Satellite orbit and clock corrections are always treated as known quantities in GPS positioning models. Therefore, any error in the satellite orbit and clock products will probably cause significant consequences for GPS positioning, especially for real-time applications. Currently three types of satellite products have been made available for real-time positioning, including the broadcast ephemeris, the International GNSS Service (IGS) predicted ultra-rapid product, and the real-time product. In this study, these three predicted/real-time satellite orbit and clock products are first evaluated with respect to the post-mission IGS final product, which demonstrates cm to m level orbit accuracies and sub-ns to ns level clock accuracies. Impacts of real-time satellite orbit and clock products on GPS point and relative positioning are then investigated using the P3 and GAMIT software packages, respectively. Numerical results show that the real-time satellite clock corrections affect the point positioning more significantly than the orbit corrections. On the contrary, only the real-time orbit corrections impact the relative positioning. Compared with the positioning solution using the IGS final product with the nominal orbit accuracy of ~2.5 cm, the real-time broadcast ephemeris with ~2 m orbit accuracy provided <2 cm relative positioning error for baselines no longer than 216 km. As for the baselines ranging from 574 to 2982 km, the cm–dm level positioning error was identified for the relative positioning solution using the broadcast ephemeris. The real-time product could result in <5 mm relative positioning accuracy for baselines within 2982 km, slightly better than the predicted ultra-rapid product.

## 1. Introduction

International GNSS Service (IGS), as a voluntary federation, has been providing the GNSS community with valuable data and products ever since 1994, including raw observation data, GNSS satellite orbit and clock products, Earth rotation parameters, and atmospheric parameters [1]. More specifically, five types of GPS satellite ephemerides are available as of 2017. The broadcast ephemeris, the IGS ultra-rapid and the real-time service products mainly aim for real-time applications, while the IGS rapid (IGR) and final (IGS) products for post-mission applications.

The GPS broadcast ephemeris is calculated by the US Air Force based on 16 worldwide monitoring stations [2]. The nominal accuracies of broadcast orbits and clocks are reported as 1 m and ~5 ns, respectively [3]. Two types of ultra-rapid products are generated by IGS, one of which is observed-half with 3~9 h latency and the other is predicted without latency. In fact, the predicted IGS ultra-rapid (hereinafter IGU) product without latency is the one utilized in most real-time or near real-time applications. The nominal IGU orbit and clock accuracies are ~5 cm and ~3 ns, respectively [3].

In order to further improve the accuracies of IGU products for real-time applications, the IGS real-time working group (RTWG) established in 2001 has coordinated the IGS real-time pilot project (RTPP) since 2007 [4]. The official IGS real-time service (RTS) was announced in April 2013, which provided corrections to GPS and GLONASS (experimental) broadcast ephemerides [5]. In 2015, an orbit accuracy of ~5 cm and a clock accuracy of ~0.3 ns with ~30 s latency for IGS real-time product have been reported by [6].

On the one hand, the broadcast ephemeris is one of the main sources for meter-level point and cm-level relative positioning. On the other hand, the IGU product has been widely adopted in near real-time relative positioning [7], meteorological [8,9,10,11,12,13,14], and timing [15] applications. Aiming at the substitution of the IGU product, the IGS real-time products have been extensively investigated during past years, in terms of real-time positioning [16,17,18,19,20,21], real-time troposphere estimation [22,23,24,25,26], and real-time seismology [27] applications.

In general, both the satellite orbit and clock products would affect positioning solutions as these two terms are normally treated as known quantities in GPS positioning models. This paper aims to study the impacts of satellite orbit and clock products on real-time GPS point and relative positioning. In Section 2, the mathematic models for GPS precise point and relative positioning are described. The real-time satellite orbit and clock products are evaluated with respect to the IGS final product in Section 3, followed by the point positioning and relative positioning analyses with different real-time satellite orbits and clock combinations in Section 4. Section 5 summarizes the impacts of satellite orbit and clock products on the real-time GPS point and relative positioning.

## 2. Mathematics

### 2.1. Point Positioning Model

In general, raw and combined observations can be adopted to conduct point positioning. For comparison purpose, the commonly used point positioning mode proposed by Zumberge et al. [28] is used in the study:

(1) $$P_{3}=\rho +c(dt^{r}-dt^{s})+d_{trop}+\varepsilon_{P_{3}}$$

(2) $$L_{3}=\rho +c(dt^{r}-dt^{s})+d_{trop}-\lambda_{3}N_{3}+\varepsilon_{L_{3}}$$

where $P_{3}=\frac{f_{1}^{2}}{f_{1}^{2}-f_{2}^{2}}P_{1}+\frac{-f_{2}^{2}}{f_{1}^{2}-f_{2}^{2}}P_{2}$ and $L_{3}=\frac{f_{1}^{2}}{f_{1}^{2}-f_{2}^{2}}L_{1}+\frac{-f_{2}^{2}}{f_{1}^{2}-f_{2}^{2}}L_{2}$ are ionosphere-free code and phase observables with $f_{1}=154f_{0},f_{2}=120f_{0},f_{0}=10.23\mathrm{MHz}$. Only the first-order ionosphere effect is removed in Equations (1) and (2), while high-order ionosphere effects are ignored. $\rho =\sqrt{{\left(X_{r}-X^{s}\right)}^{2}+{\left(Y_{r}-Y^{s}\right)}^{2}+{\left(Z_{r}-Z^{s}\right)}^{2}}$ is the geometric distance between the receiver with coordinates $\left(X_{r},Y_{r},Z_{r}\right)$ and the satellite with coordinates $\left(X^{s},Y^{s},Z^{s}\right)$; *c* is the speed of light in vacuum; $dt^{r}$ is the receiver clock error; $dt^{s}$ is the satellite clock error; $d_{trop}$ is the troposphere effect which can be dealt with by correcting the troposphere zenith hydrostatic part and estimating the troposphere zenith wet part; $\lambda_{3}$ is the carrier phase wavelength; $N_{3}$ is the phase ambiguity; and $\varepsilon_{*}$ is the residual error containing multipath and observation noise. It should be noted that the ambiguity parameter in Equation (2) is real-valued, rather than integer-valued, as the code and phase biases are not considered in this traditional PPP model [29].

Regarding the geometric distance *ρ*, the satellite coordinates, or the satellite orbits, are always fixed as known quantities. The satellite clock products are directly used to remove the satellite clock error $dt^{s}$ in the PPP model. Unknown parameters in the GPS point positioning model include the receiver coordinates, the receiver clock error, the troposphere zenith wet delay, and the phase ambiguities. 

### 2.2. Relative Positioning Model

The function model for GPS relative positioning is:

(3) $$\Delta \nabla P_{3}=\Delta \nabla \rho +\Delta \nabla d_{trop}+\varepsilon_{\Delta \nabla P_{3}}$$

(4) $$\Delta \nabla L_{3}=\Delta \nabla \rho +\Delta \nabla d_{trop}-\lambda_{3}\Delta \nabla N_{3}+\varepsilon_{\Delta \nabla L_{3}}$$

where Δ∇ is the double-differencing operator which indicates differencing between satellites and receivers. For example, $\Delta \nabla P_{3}=({P_{3}}_{r1}^{i}-{P_{3}}_{r2}^{i})-({P_{3}}_{r1}^{j}-{P_{3}}_{r2}^{j})$ with subscripts r1,r2 as two receivers and superscripts i,j as two satellites. All other terms have the same meaning as those in Equations (1) and (2).

Unlike the point positioning model, the relative positioning model removes both the satellite and receiver clock errors. The satellite orbit, however, still remains in the double-differenced geometric distance Δ∇ρ, which would subsequently affect the parameter estimation. The unknown parameters in the relative positioning model include the receiver coordinates, the troposphere zenith wet delay, and the double-differenced phase ambiguities. Depending on the distance between receivers, the troposphere parameters can be estimated as one relative troposphere delay (for short baselines) or two absolute troposphere delays (for long baselines).

## 3. Satellite Orbit and Clock Accuracy

As mentioned in the Introduction section, there are three types of real-time satellite orbits and clock ephemeris, or products, i.e., the broadcast ephemeris (hereinafter BRDC) which is transmitted in the GPS satellite navigation message, the IGU product which can also be freely retrieved via the Internet, and the IGS real-time product available for registered users at no economic costs. In addition to these three real-time products, another two types of precise orbit and clock products are also obtainable, but mainly for post-mission applications, namely the IGR product with >17 h latency and the IGS final product with >12 days latency. In this section, the IGS final product with a nominal orbit accuracy of ~2.5 cm and a clock accuracy of ~0.075 ns is selected as a reference to assess the accuracies of the three real-time satellite products. The IGR orbit and clock products are also included to conduct the performance comparison among the real-time and post-mission products.

As the IGU products are updated every six hours (four times per day), only the most recent six-hour orbit and clock corrections are used in this study. Once the new IGU products are available, the most recent IGU corrections are used and the previous IGU products beyond six hours are discarded. In this way, the maximum predicted interval of the IGU products that can be controlled is six hours.

The IGS RTS currently provides three combined real-time satellite orbit and clock products based on contributions from several participating agencies. In the meantime, the real-time satellite corrections from each participating agency are also available for registered users. The IGS-RTS CLK51 stream generated by CNES (hereinafter CNT) is chosen in this study because of its high stability of satellite clock corrections compared with other IGS real-time correction streams [30].

**Figure 1:**
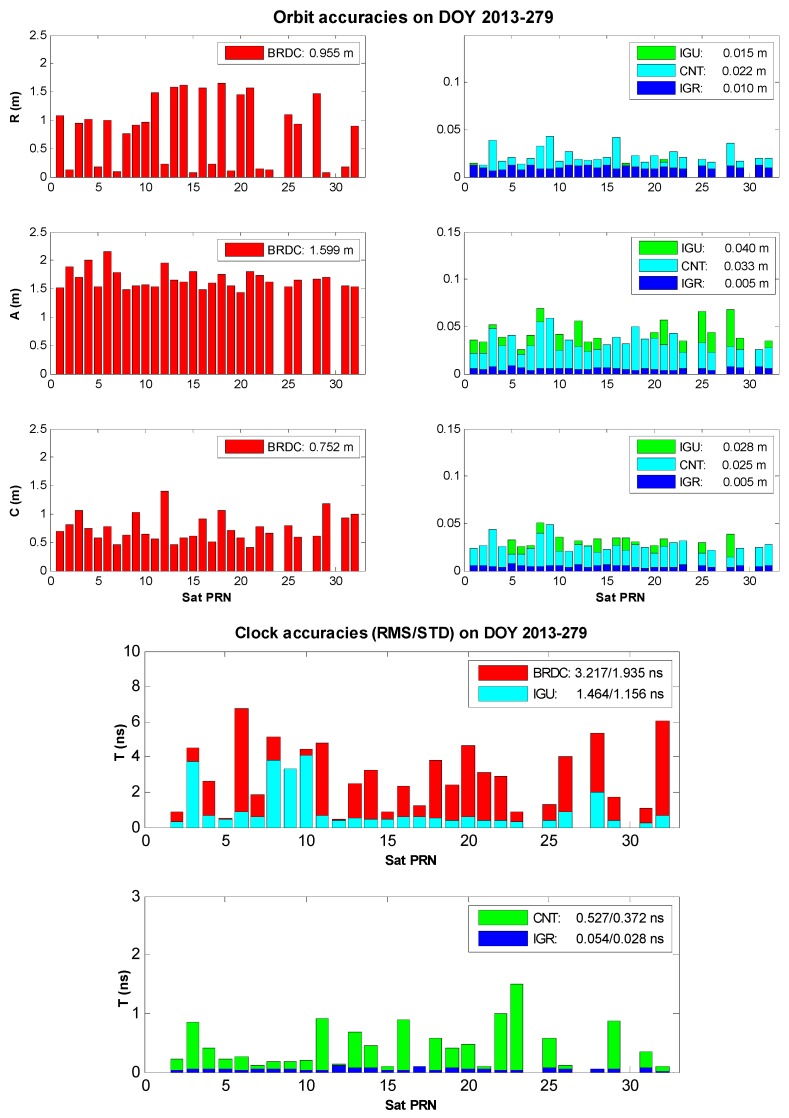
Orbit (**top**) and clock (**bottom**) accuracies of BRDC, IGU, CNT, and IGR satellite products with respect to the IGS final product on DOY 2013-279.

The orbit and clock accuracies with respect to the IGS final product over the day of year (DOY) 2013-279 are illustrated in Figure 1. At first, the BRDC orbit is obviously worse than the other three counterparts. The BRDC orbit accuracy in each component ranges from 0.752 to 1.839 m while accuracies of the others are at the level of mm to cm. Second, the IGU orbit is slightly worse than the CNT orbit, but the difference is not significant. The 3D IGU orbit accuracy is 0.054 m and that of CNT orbit is 0.047 m. Third, the IGR orbit, the sole post-mission product among the comparison, possesses the best orbit accuracy of <1 cm.

Regarding the satellite clock comparison, both the root mean square (RMS) and the standard deviation (STD) are adopted to evaluate the involved clock products. As the common part of the satellite clock errors can be absorbed by the receiver clock parameter, the satellite clock STD is more important than the clock RMS for point positioning applications. Given that the reference clock (IGS final product) is tabulated at an interval of 15 min, all the intervals of BRDC, IGU, CNT, and IGR clocks are reset as 15 min in order to avoid possible satellite clock interpolation errors. The satellite with pseudo-range noise (PRN) #1 is selected as the reference satellite in all clock products. The other non-reference satellite clocks are differenced with the reference satellite clock to remove the clock datum inconsistency among the clock products for comparison [19]. If the clock correction of one certain satellite in any satellite clock product is abnormal or missing, this satellite is removed from the comparison, such as satellites PRN #24, #27, and #30 in Figure 1.

First, the BRDC clock suffers the largest errors of 3.217/1.935 ns RMS/STD. Second, the IGU clock is evaluated with the RMS of 1.464 ns and the STD of 1.156 ns. Although the IGU clock is more precise than the BRDC clock, both the IGU and BRDC clock errors are much larger than the other clocks by at least one-order of magnitude. This phenomenon is quite different from the orbit case that IGU orbit is comparative to the CNT and IGR orbits. Third, the CNT clock with the RMS of 0.527 ns and the STD of 0.372 ns is much more precise than BRDC and IGU clocks, but worse than the IGR clock whose RMS is 0.054 ns and STD is 0.028 ns.

Table 1 presents the satellite orbit and clock accuracies over the period from DOY 2013 279 to 285. The overall orbit accuracies are 2.203/0.047/0.045/0.012 m for the BRDC/IGU/CNT/IGR products, respectively. With regards to the overall clock accuracies, the RMS values of 3.550/1.516/0.550/0.071 ns and the STD values of 2.360/1.158/0.383/0.050 ns are obtained for the BRDC/IGU/CNT/IGR products, respectively. These four types of satellite products with different magnitudes of the orbit and clock errors contribute to the analysis of the satellite orbit and clock impacts on the real-time point and relative positioning in the following content.

## 4. Experiment and Analysis

### 4.1. Experiment Description

**Figure 2:**
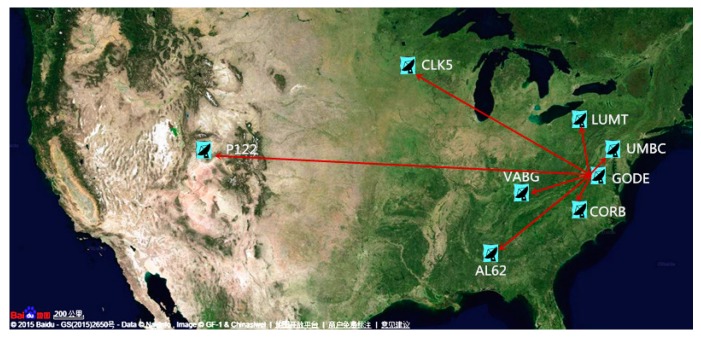
Selected GPS stations.

In order to assess the real-time satellite orbit and clock effects on point and relative positioning, eight GPS tracking stations from the National Geodetic Survey (NGS) Continuously Operating Reference Station (CORS) network [31] are selected in this study (Figure 2). The approximate location and the receiver/antenna types are given in Table 2. On the one hand, the point positioning assessment is conducted by processing raw GPS observations of the eight stations using The University of Calgary’s software package P3 [32]. On the other hand, seven baselines ranging from 28 to 2982 km (Table 3) are processed using the GAMIT software package to exploit the real-time satellite orbit and clock effects on relative positioning. Both the point and relative positioning is performed in the static mode with an observation sampling rate of 30 s.

Three processing strategies (PS) with various real-time orbits and clock combinations are presented in Table 4. The latencies of the orbit and clock corrections are depicted in the parenthesis. All satellite orbits in the PS #1 are fixed as the IGS final orbit, so this strategy focuses on the effects of various clock products on positioning. In the meantime, the PS #2 fixes all clocks as the IGS final clock so that the effects of various orbit products can be studied. It should be noted that the consistency within the orbit and clock product should be maintained as some orbit errors can be absorbed by the consistent clock error [33]. That is to say, the effect of the inconsistency between the utilized orbit and the clock products has not been concerned in the first two processing strategies. Therefore, the PS #3 concerning the consistency between the satellite orbits and clocks is designed to investigate the combined effects of real-time satellite orbit and clock products on practical positioning applications.

### 4.2. Point Positioning

The PPP coordinates using IGS final products are served as reference. Since the BRDC orbit and clock errors are too large to obtain precise point coordinates, the BRDC product is not concerned in this analysis. Only the IGU, CNT, and IGR products are investigated for the point positioning analysis. The horizontal and vertical errors are computed as the RMS of the last ten-minute coordinate series of the daily coordinate solution.

**Figure 3:**
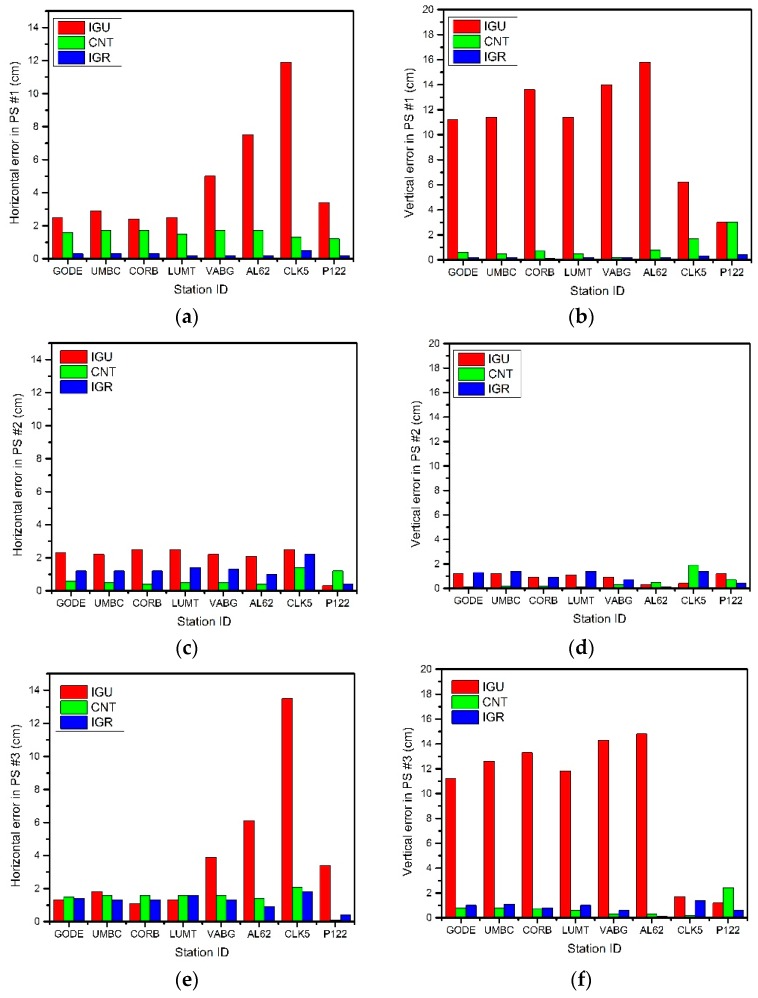
Horizontal (**a,c,e**) and vertical (**b,d,f**) point positioning errors of the PS #1 (**a,b**), #2 (**c,d**), and #3 (**e,f**) on DOY 279.

As for the PS #1 with common orbit corrections and different clock corrections, the IGU coordinate solutions are obviously worse than the CNT and the IGR solutions in Figure 3. The point positioning model in Equations (1) and (2) demonstrates that both the satellite orbit and the clock corrections would affect the point positioning. Since the satellite orbit product is fixed for all of the positioning solutions, it could then be concluded that the IGU clock product of 1.156 ns STD results in cm- to dm-level horizontal and vertical positioning errors. The CNT clock product of 0.372 ns STD causes <3 cm horizontal and vertical errors while only mm-level positioning errors are identified for the IGR clock product with the STD of 0.028 ns.

In PS #2 where all clock corrections are fixed as the IGS final clock product, the effects of various orbit corrections are found very limited for the point positioning solutions. More specifically, 0~3 cm positioning accuracies are identified for all of the IGU, CNT, and IGR coordinate solutions.

The practical combined effects of real-time satellite orbit and clock products on point positioning are assessed by PS #3, and the results are depicted in the bottom row in Figure 3. The overall performance of PS #3 coordinate solutions is quite similar to that of PS #1, which indicates the satellite clock corrections play a more crucial role than the orbit corrections for point positioning. The slight difference between the PS #3 and the PS #1 coordinate solutions can be attributed to the inconsistency within the satellite orbit and clock corrections as PS #1 adopts mixed satellite orbit and clock products.

All in all, numerical results shown in Figure 3 demonstrate that the effect of real-time satellite clock corrections is more significant than that of the real-time satellite orbit corrections for GPS point positioning.

### 4.3. Relative Positioning

Unlike the ignorance of the BRDC ephemeris in the point positioning analysis, the BRDC satellite ephemeris is included in this sub-section as it is one widely used satellite product for relative positioning. The baseline is calculated using daily observations of two receivers. The horizontal and vertical errors are computed as the difference of the calculated baseline with respect to the reference baseline using the IGS final products.

**Figure 4:**
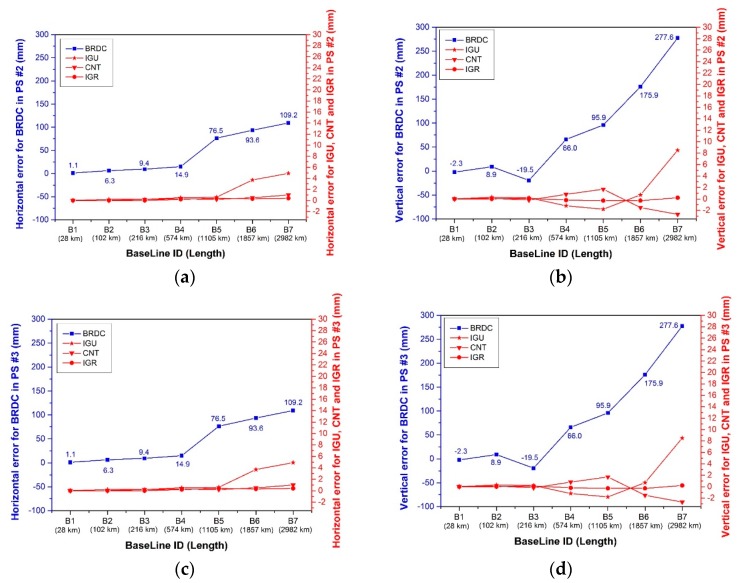
Horizontal (**a,c**) and vertical (**b,d**) relative positioning errors of the PS #2 (**a,b**) and #3 (**c,d**) on DOY 279.

Since the satellite clock errors are eliminated in the relative positioning model as shown in Equations (3) and (4), all horizontal and vertical errors in PS #1 are zero, so no plot is made for PS #1. It is clear from the top plots in Figure 4 that once various satellite orbit products are involved, the relative positioning results present quite different tendency in PS #2. First, both the horizontal and vertical positioning errors of the IGR solution is less than 1 mm for all baselines. Second, the CNT solution with ~5 cm orbit accuracy demonstrates very good performance in the horizontal component. With the baseline length increasing to 2982 km, ~2 mm vertical errors are identified. Third, mm level horizontal positioning accuracy can also be obtained using the IGU orbit product. A distance-dependent pattern has been identified for baselines longer than 1105 km. Additionally, the vertical accuracy is good within 1 mm for baselines no longer than 216 km. When the baseline length increases, the vertical positioning error degrades from less than 1 mm to 8 mm. Fourth, the BRDC product with 2.203 m orbit accuracy yields the worst relative positioning solution. Positioning errors at mm- to cm- levels are achievable for baselines no longer than 216 km, whereas sub-dm- to dm-level errors are obtained for baselines beyond 216 km. The distance-dependent baseline error pattern agrees well with the rule of thumb described in Chapter 5.01 of Wells et al. [34].

As the satellite clock term is removed and the satellite orbit is treated as a known quantity in the relative model, the effect caused by the inconsistency between the satellite orbit and clock products can be negligible. Therefore, the positioning errors in PS #3 are the same as those in PS #2.

Table 5 tabulates the horizontal and vertical positioning errors over the period from DOY 2013 279 to 285 for PS #3. The phenomena identified in the daily analysis can be further confirmed by the weekly analysis. The satellite clock corrections do not impact the relative positioning at all as all coordinate differences are calculated as zero (not listed in the manuscript). On the contrary, the satellite orbit corrections affect relative positioning more significantly. The CNT product provides slight better relative positioning accuracy than the IGU product at the level of sub-mm to mm. The BRDC positioning errors show apparent distance-dependent patterns, and are significantly larger than the other products. The cm-level relative errors are obtained for baselines no longer than 216 km, and cm- to dm-level errors for baselines beyond 216 km. It should be noted that the inconsistency issue of satellite orbit and clock products which are observed to be un-negligible for point positioning do not cause significant effects for the relative positioning, since only sub-mm differences are detected in the PS #2 for two out of 196 baselines.

## 5. Conclusions

Since the satellite orbit and clock corrections are always treated as known quantities in GPS positioning models, any error of satellite orbit and clock products would probably cause significant consequences on positioning solutions, especially for real-time positioning. As such, it is of great value to investigate the effect of real-time satellite orbit and clock products on the two well-known GPS positioning techniques, namely the point positioning and the relative positioning.

This paper investigates the effect of three satellite orbit and clock products on both the real-time point and relative positioning. The BRDC ephemeris possesses the 3D orbit accuracy of ~2 m and the clock RMS/STD accuracies of 3.550/2.360 ns. The IGU and the CNT orbits are approximate to each other at the level of ~5 cm, whereas the IGU clock with the RMS/STD accuracies of 1.516/1.158 ns is much worse than the CNT clock of 0.550/0.383 ns. Using these three real-time satellite products, different characteristics are summarized for the two positioning techniques. As for the point positioning, the real-time satellite clock product plays a much more important role than the orbit product. On the contrary, the real-time satellite orbit product affects the relative positioning more significantly.

It can also be concluded that the CNT real-time satellite orbit and clock products can provide mm- to cm-level point and mm-level relative positioning solutions compared with the post-mission IGS counterparts. The IGU real-time product with relatively worse clock accuracy can yield cm- to dm-level point and mm-level relative positioning accuracies. As the most commonly used orbit and clock corrections, the BRDC ephemeris with ~2 m orbit and ~3 ns clock errors are able to provide mm-to dm-level relative positioning accuracies depending on the baseline length between the receivers.

Table 6 summarizes the positioning performance of various satellite products in terms of positioning accuracy and the feasibility for real-time applications. As the new product, the IGS real-time product with CNT as the representative can fill the gap between the existing IGR product and the IGU product. More specifically, CNT can provide comparable point positioning accuracy as IGR, but with no latency in practice. Moreover, CNT can provide better relative positioning accuracy than IGU. Therefore, the IGS real-time product can benefit the high-accurate real-time point positioning, and also the real-time relative positioning, even for >2000 km baselines, in practical GPS positioning applications.

## Figures and Tables

**Table 1:** Satellite orbit and clock accuracies over the period from DOY 2013 279 to 285.

DOY	Satellite Product	Orbit (m)				Clock (ns)	
		R	A	C	3D	RMS	STD
279	BRDC	0.955	1.839	0.752	2.205	3.217	1.935
	IGU	0.015	0.040	0.028	0.054	1.469	1.145
	CNT	0.022	0.033	0.025	0.047	0.527	0.372
	IGR	0.01	0.005	0.005	0.012	0.054	0.028
280	BRDC	0.962	1.823	0.734	2.195	3.578	2.324
	IGU	0.016	0.037	0.023	0.049	1.990	1.615
	CNT	0.025	0.032	0.024	0.047	0.520	0.342
	IGR	0.009	0.005	0.005	0.012	0.069	0.039
281	BRDC	0.972	1.870	0.696	2.207	3.150	2.061
	IGU	0.014	0.039	0.027	0.048	1.225	0.927
	CNT	0.023	0.030	0.025	0.043	0.514	0.349
	IGR	0.009	0.005	0.005	0.012	0.061	0.038
282	BRDC	0.963	1.862	0.653	2.130	3.746	2.665
	IGU	0.013	0.038	0.026	0.048	1.561	1.198
	CNT	0.026	0.031	0.024	0.046	0.622	0.437
	IGR	0.009	0.006	0.006	0.012	0.080	0.071
283	BRDC	0.970	1.874	0.648	2.226	3.407	2.353
	IGU	0.013	0.031	0.020	0.039	1.189	0.873
	CNT	0.023	0.031	0.026	0.046	0.530	0.353
	IGR	0.009	0.006	0.006	0.013	0.067	0.035
284	BRDC	0.975	1.881	0.613	2.223	3.862	2.628
	IGU	0.011	0.035	0.021	0.042	1.371	0.998
	CNT	0.021	0.029	0.021	0.042	0.539	0.365
	IGR	0.009	0.006	0.006	0.012	0.076	0.046
285	BRDC	0.963	1.855	0.653	2.232	3.807	2.445
	IGU	0.013	0.034	0.026	0.046	1.635	1.166
	CNT	0.019	0.029	0.022	0.042	0.587	0.445
	IGR	0.009	0.006	0.005	0.013	0.085	0.069
**Overall**	**BRDC**	**0.966**	**1.858**	**0.678**	**2.203**	**3.550**	**2.360**
	**IGU**	**0.014**	**0.036**	**0.024**	**0.047**	**1.516**	**1.158**
	**CNT**	**0.023**	**0.031**	**0.024**	**0.045**	**0.550**	**0.383**
	**IGR**	**0.009**	**0.006**	**0.005**	**0.012**	**0.071**	**0.050**

**Table 2:** Station information.

Site ID	Lat (Degree)	Lon (Degree)	Receiver Type	Antenna Type
GODE	39.0217	−76.8267	ASHTECH UZ-12	AOAD/M_T
UMBC	39.2567	−76.7114	TRIMBLE NETR9	TRM57971
CORB	38.2019	−77.3733	LEICA GRX1200PRO	ASH700936E
LUMT	40.6014	−75.3575	TRIMBLE NETRS	TRM29659
VABG	36.9323	−82.6831	TRIMBLE NETRS	TRM41249
AL62	32.1481	−85.6867	LEICAGRX1200GGPRO	LEIAX1202GG
CLK5	44.9356	−97.9606	ASHTECH Z-XII3	TRM41249USCG
P122	41.6353	−112.3317	TRIMBLE NETRS	TRM29659

**Table 3:** Baseline information.

Baseline ID	Starting Point	Ending Point	Baseline Length (km)
B1	GODE	UMBC	28
B2	GODE	CORB	102
B3	GODE	LUMT	216
B4	GODE	VABG	574
B5	GODE	AL62	1105
B6	GODE	CLK5	1857
B7	GODE	P122	2982

**Table 4:** Real-time satellite orbit and clock combination in three processing strategies.

Strategy #	Satellite Product	Orbit (Latency)	Clock (Latency)
1	BRDC	IGS final (>12 days)	BRDC (0)
	IGU		IGU (0)
	CNT		CNT (0)
	IGR		IGR (>17 h)
2	BRDC	BRDC (0)	IGS final (>12 days)
	IGU	IGU (0)	
	CNT	CNT (0)	
	IGR	IGR (>17 h)	
3	BRDC	BRDC (0)	BRDC (0)
	IGU	IGU (0)	IGU (0)
	CNT	CNT (0)	CNT (0)
	IGR	IGR (>17 h)	IGR (>17 h)

**Table 5:** Horizontal/vertical relative positioning errors (mm) in PS #3 over the period from DOY 2013 279 to 285.

DOY	Satellite Product	Baseline ID (Length)						
		B1 (28 km)	B2 (102 km)	B3 (216 km)	B4 (574 km)	B5 (1105 km)	B6 (1857 km)	B7 (2982 km)
279	BRDC	1.1/−2.3	6.3/8.9	9.4/−19.5	14.9/66.0	76.5/95.9	93.6/175.9	109.2/277.6
	IGU	0.0/0.0	0.2/0.3	0.2/0.2	0.5/−1.2	0.6/−1.8	3.7/0.7	4.9/8.5
	CNT	0.0/0.0	0.0/0.1	0.2/−0.2	0.3/0.8	0.2/1.7	0.5/−1.5	1.0/−2.7
	IGR	0.0/0.0	0.0/0.0	0.0/0.1	0.2/−0.2	0.4/−0.3	0.3/−0.3	0.4/0.2
280	BRDC	1.9/−5.1	7.3/7.9	12.4/−15.3	18.4/51.1	65.8/83.4	97.1/174.3	118.2/236.8
	IGU	0.0/0.0	0.1/0.0	0.2/0.1	0.3/−1.0	0.6/1.1	1.2/1.1	2.5/3.5
	CNT	0.0/0.0	0.0/0.1	0.1/−0.3	0.2/0.8	0.1/2.2	2.1/−0.2	1.1/−3.5
	IGR	0.0/0.0	0.0/0.0	0.0/0.0	0.1/−0.1	0.2/−0.2	0.1/0.2	0.2/−0.3
281	BRDC	1.3/−2.7	4.9/9.7	17.5/−19.7	17.9/61.2	64.9/92.9	110.0/145.0	125.1/225.9
	IGU	0.0/0.1	0.0/−0.2	0.1/0.6	0.4/−1.3	1.2/−3.0	1.0/−2.9	0.7/−3.5
	CNT	0.0/0.0	0.0/0.1	0.1/−0.2	0.0/0.8	0.1/1.6	0.2/−0.1	1.1/1.3
	IGR	0.0/0.0	0.0/0.0	0.0/0.1	0.2/−0.2	0.4/−0.3	0.5/−0.2	0.7/−0.1
282	BRDC	1.1/−3.2	6.6/2.4	20.3/−13.7	32.8/59.6	82.9/103.0	97.2/211.0	66.7/358.5
	IGU	0.0/0.0	0.0/0.0	0.1/0.0	0.4/−0.5	0.4/−0.6	1.5/−3.1	1.5/−2.3
	CNT	0.0/0.0	0.0/0.0	0.2/−0.1	0.3/0.3	0.6/0.7	1.1/−1.8	1.4/−2.3
	IGR	0.0/0.0	0.0/0.0	0.0/0.1	0.1/−0.2	0.2/−0.2	0.4/−0.3	0.5/0.1
283	BRDC	1.5/−3.3	3.0/4.0	22.0/−17.7	30.6/74.7	103.7/124.0	114.3/185.8	100.8/298.0
	IGU	0.0/0.0	0.1/0.1	0.2/−0.1	0.4/−0.4	1.3/−0.6	0.5/−0.5	2.5/−2.6
	CNT	0.0/0.0	0.0/0.1	0.0/−0.1	0.3/0.3	0.6/0.2	0.5/−0.3	0.8/0.4
	IGR	0.0/0.0	0.0/0.0	0.0/0.0	0.1/−0.1	0.4/−0.6	0.5/−0.2	0.4/0.4
284	BRDC	1.0/−3.1	6.7/7.9	17.4/−16.8	31.8/77.0	85.9/123.3	137.5/225.9	198.0/294.1
	IGU	0.0/0.0	0.1/0.1	0.1/−0.1	0.3/0.6	0.6/1.0	0.9/0.7	1.0-0.4
	CNT	0.0/0.0	0.1/0.1	0.2/−0.1	0.3/0.4	0.8/1.1	0.5/−0.2	0.4/−1.5
	IGR	0.0/0.0	0.0/0.0	0.0/0.1	0.2/−0.1	0.3/0.0	0.4/0.1	0.7/−0.3
285	BRDC	0.9/−2.8	7.5/10.2	29.1/−17.6	31.9/66.8	105.6/120.7	106.7/195.0	142.1/278.0
	IGU	0.0/0.0	0.1/0.1	0.2/−0.2	0.7/1.0	0.6/2.0	2.1/2.2	3.2/5.4
	CNT	0.0/0.0	0.1/0.1	0.2/0.0	0.4/1.1	0.6/1.0	1.1/2.7	0.8/5.4
	IGR	0.0/0.0	0.0/0.1	0.0/0.0	0.2/0.0	0.2/0.2	0.4/0.6	0.6/0.6
**Overall**	**BRDC**	**1.3/3.3**	**6.2/7.8**	**19.3/17.3**	**26.5/65.7**	**85.0/107.3**	**109.0/189.2**	**128.5/284.2**
	**IGU**	**0.0/0.0**	**0.1/0.2**	**0.2/0.3**	**0.4/0.9**	**0.8/1.7**	**1.8/1.9**	**2.7/4.4**
	**CNT**	**0.0/0.0**	**0.1/0.1**	**0.2/0.2**	**0.3/0.7**	**0.5/1.4**	**1.0/1.4**	**1.0/2.9**
	**IGR**	**0.0/0.0**	**0.0/0.0**	**0.0/0.1**	**0.2/0.1**	**0.3/0.3**	**0.4/0.3**	**0.5/0.3**

**Table 6:** Positioning performance of various satellite products in terms of positioning accuracy and the feasibility for real-time applications.

	Point		Relative		
	Accuracy	Real-Time	Accuracy		Real-Time
			Baseline <200 km	Baseline >2000 km	
BRDC	× *	√	2	×	√
IGU	3	√	1	3	√
CNT	2	√	1	2	
IGR	1 **	×	1	1	×

* × means inapplicable; √ means applicable. ** 1, 2, 3 means the positioning accuracy level. 1: the best accuracy; 3: the worst accuracy.
